# Arbutin attenuates nephrotoxicity induced by gentamicin

**Published:** 2021

**Authors:** Elnaz Emadi, Mahdi Pouramir, Maryam Ghasemi-Kasman, Farideh Feizi, Sohrab Halalkhor, Ali Akbar Moghadamnia

**Affiliations:** 1 *Student Research Committee, Babol University of Medical Sciences, Babol, Iran*; 2 *Department of Clinical Biochemistry, Faculty of Medicine, Babol University of Medical Sciences, Babol, Iran*; 3 *Cellular and Molecular Biology Research Center, Health Research Institute, Babol University of Medical Sciences, Babol, Iran*; 4 *Department of Anatomy, Faculty of Medicine, Babol University of Medical Sciences, Babol, Iran*; 5 *Neuroscience Research Center, Health Research Institute, Babol University of Medical Sciences, Babol, Iran*; 6 *Department of Pharmacology, Faculty of Medicine, Babol University of Medical Sciences, Babol, Iran*

**Keywords:** Gentamicin, Nephrotoxicity, Arbutin, Antioxidant, Histopathology

## Abstract

**Objective:**

In this study, the impact of arbutin was examined in a gentamicin (GM)-induced nephrotoxicity model.

**Materials and Methods:**

Forty adult male Wistar rats were randomly assigned to five groups including control group; GM group, and three groups of GM+arbutin (25, 50 and 75 mg/kg). One day after the last injection of GM, creatinine, urea, carbonyl, thiobarbituric acid-reacting substance (TBARs), ferric reducing antioxidant power (FRAP) and 8-hydroxyguanosine levels were assessed in serum samples. Left and right kidneys were used for biochemical assays and histological evaluation, respectively.

**Results:**

Our data showed that the FRAP level (p<0.05), urea (p<0.001), creatinine (p<0.001), and 8-hydroxyguanosine (p<0.001) levels of serum samples, were increased in GM-treated rats compared to the controls. The serum levels of TBARS (p<0.001) and carbonyl increased in serum and renal tissue (p<0.001) of GM-treated animals. Conversely, arbutin attenuated serum creatinine, urea and 8-hydroxyguanosine, and TBARS (p<0.001). Administration of arbutin significantly decreased carbonyl levels in serum and renal tissue samples (p<0.001). Furthermore, the levels of FRAP increased in the serum (p<0.01) and renal tissue samples (p<0.001) of arbutin-treated animals. Histological staining showed that arbutin significantly inhibits kidney damages.

**Conclusion:**

Our data suggest that arbutin attenuates GM-induced nephrotoxicity through its free radicals-scavenging activity.

## Introduction

Aminoglycoside antibiotics are often applied to treat severe infections of the urinary tract and abdomen (Nagai and Takano, 2004 ▶). Gentamicin (GM) as an aminoglycoside antibiotic is routinely administrated for treating Gram-negative bacterial infections (Cao et al., 2019 ▶). GM causes functional, metabolic and morphologic changes in the kidney (Mingeot-Leclercq et al., 1999 ▶). It also generates reactive oxygen species (ROS), increases the level of lipid peroxidation (LPO), and reduces the antioxidant enzymes activities in the kidney and intestine (Farooq et al., 2007 ▶; Banday et al., 2008 ▶). However, the mechanism of GM-induced nephrotoxicity has not been fully known (Ali, 2003 ▶), but oxidative stress has a major role in GM-induced toxicity (Cao et al., 2019 ▶). 

It has been shown that natural products with antioxidant activity considerably suppress or improve the GM-induced nephrotoxicity (Boroushaki and Sadeghnia, 2009 ▶; Hasanvand et al., 2018 ▶; Boroushaki et al., 2019 ▶; Cao et al., 2019 ▶). 

*Pyrus boissieriana* from Rosaceae family is regarded as one of the main natural resources of arbutin (Shahaboddin et al., 2011 ▶). Arbutin possesses various therapeutic and pharmacological properties such as antioxidant, anti-inflammatory, anti-hyperlipidemic, antiviral, free radical scavenging, anti-hyperglycemic, and gastroprotective activities (Capasso et al., 2007 ▶; Shahaboddin et al., 2011 ▶; Taha et al., 2012 ▶; Yousefi et al., 2013 ▶). Our recent studies also demonstrated that arbutin has beneficial effects in Alzheimer's disease (AD) (Dastan et al., 2019 ▶), epilepsy (Ahmadian et al., 2019 ▶) and Parkinson's disease (PD) (Dadgar et al., 2018 ▶).

In this study, the possible protective effect of arbutin against GM nephrotoxicity was examined using biochemical assays and histopathological evaluation of kidneys in rats.

## Materials and Methods

**Chemicals**

Arbutin (>96% purity) and GM were obtained from Fluka (Switzerland) and Caspian Tamin Pharmaceutical Company (Iran), respectively. 8-hydroxyguanosine and protein carbonyl kits were purchased from ZellBio GmbH (Germany). Thiobarbituric acid (TBA), absolute ethanol and 2, 4, 6-tripyridyl-s-triazine (TPTZ) were provided from Merck Company (Germany). 

**Animals**

This experimental study was done on 40 male Wistar rats weighing 150-200 g. The rats were kept under standard laboratory conditions with 12 hr light/12 hr dark cycle. The rats were fed with normal chow and drinking water. All procedures were approved by the ethics committee of Babol University of Medical Sciences.

**Experimental design**

Totally, 40 adult male Wistar rats were randomly assigned to five groups (n=8 in each group): (1) control group: the rats received a daily intraperitoneal (i.p.) injection of saline (0.5 ml/kg) for 8 days; (2) GM group: 100 mg/kg GM was i.p. injected for 8 days (Farombi and Ekor, 2006 ▶) and Groups 3-5: the rats received i.p. injections of arbutin (25, 50 or 75 mg/kg, respectively) (Khadir et al., 2015 ▶), 1 hr after the injection of GM for 8 days. In order to prepare the appropriate dosage of arbutin, it was dissolved in sterile normal saline. 

**Biochemical assessment**

Rats were anesthetized by ether and blood was collected from the axillary artery. Then, serum was separated and used for assessment of creatinine, urea, carbonyl, thiobarbituric acid reactive substances (TBARS), and ferric reducing ability of plasma (FRAP). After blood sampling, rats were sacrificed and the left kidney was immediately, removed, and used for assessment of FRAP, TBARS, carbonyl, and 8-hydroxiguanosine levels (Khadir et al., 2015 ▶; Dadgar et al., 2018 ▶; Dastan et al., 2019 ▶). 

**Histopathological evaluation**

After anesthetizing using ether, the right kidney was removed and fixed in 10% formalin. Serial sections (5 µm) were prepared using microtome and stained with hematoxylin and eosin stain (H&E). After preparation and staining of the tissue samples, images from each tissue section were randomly taken from 4 regions using Canon camera (Canon, pc1587. JAPAN) attached to an optical microscope (Olympus, Japan).

**Statistical analysis**

GraphPad prism 6 software (GraphPad software Inc. San Diego, CA, USA) was used for analysis of the data. The results were assessed by one-way analysis of variance (ANOVA) and Tukey *post hoc*. Experimental data are expressed as mean± SEM and p<0.05 was considered statistically significant. 

## Results

**Effects of arbutin on urea, creatinine, carbonyl, and 8-hydroxyguanosine levels in GM-treated rats**

**Figure 1:**
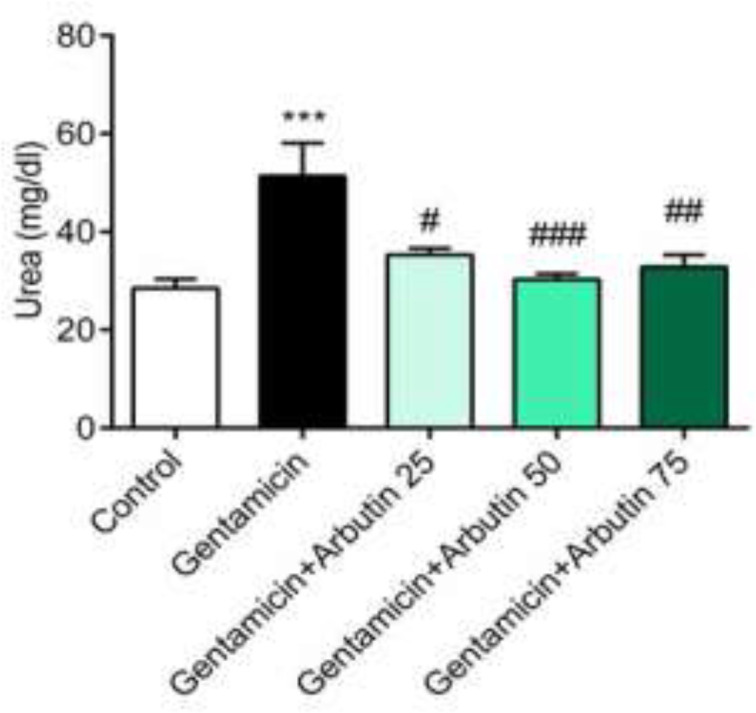
Effect of arbutin on urea level in GM-induced nephrotoxicity

^***^p<0.001 compared to the control; ^#^p<0.05, ^##^p<0.01 and ^###^p<0.001 compared to the GM group. n=8. GM: Gentamicin.

The results illustrated that serum urea level in the group receiving GM was markedly increased compared to the control rats (p<0.001). Urea level was higher in group receiving GM plus arbutin at dose of 50 mg/kg compared to the GM group (p<0.001). However, in comparison to the GM-treated rats, the difference was lower for the groups receiving GM with arbutin at doses of 25 (p<0.05) and 75 mg/kg (p<0.01) (Figure 1).

**Figure 2:**
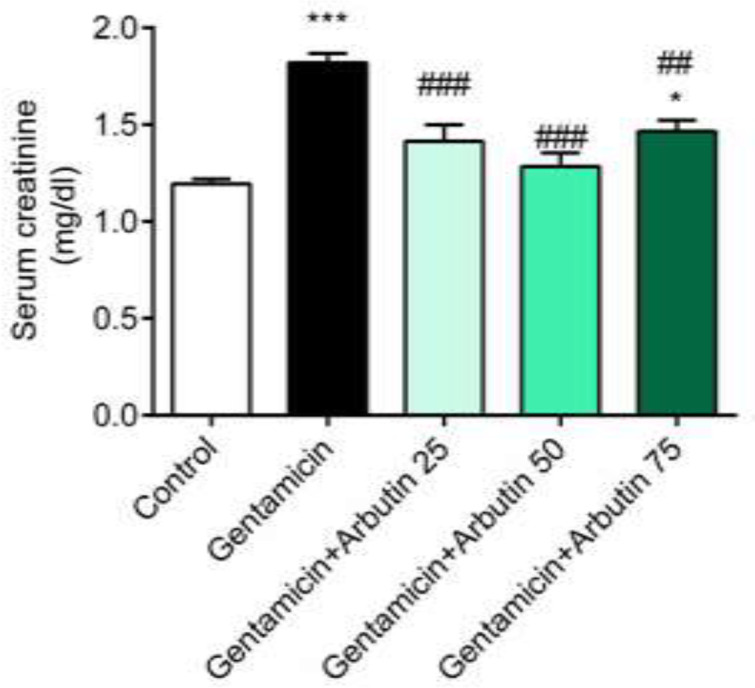
Effect of arbutin on creatinine level in GM-induced nephrotoxicity

^*^p<0.05 and^ ***^p<0.001 compared to the control; ^##^p<0.01 and ^###^p<0.001 compared to the GM group. n=8. GM: Gentamicin.

The level of creatinine significantly increased in GM-receiving rats (p<0.001) and GM+arbutin (75 mg/kg) (p<0.05) compared to the control. Additionally, the creatinine level was significantly decreased in the arbutin-treated rats (for arbutin 25 mg/kg, p< 0.001); for arbutin 50 mg/kg, p<0.001, and for arbutin 75 mg/kg, p<0.001) compared to the GM group (Figure 2).

**Figure 3:**
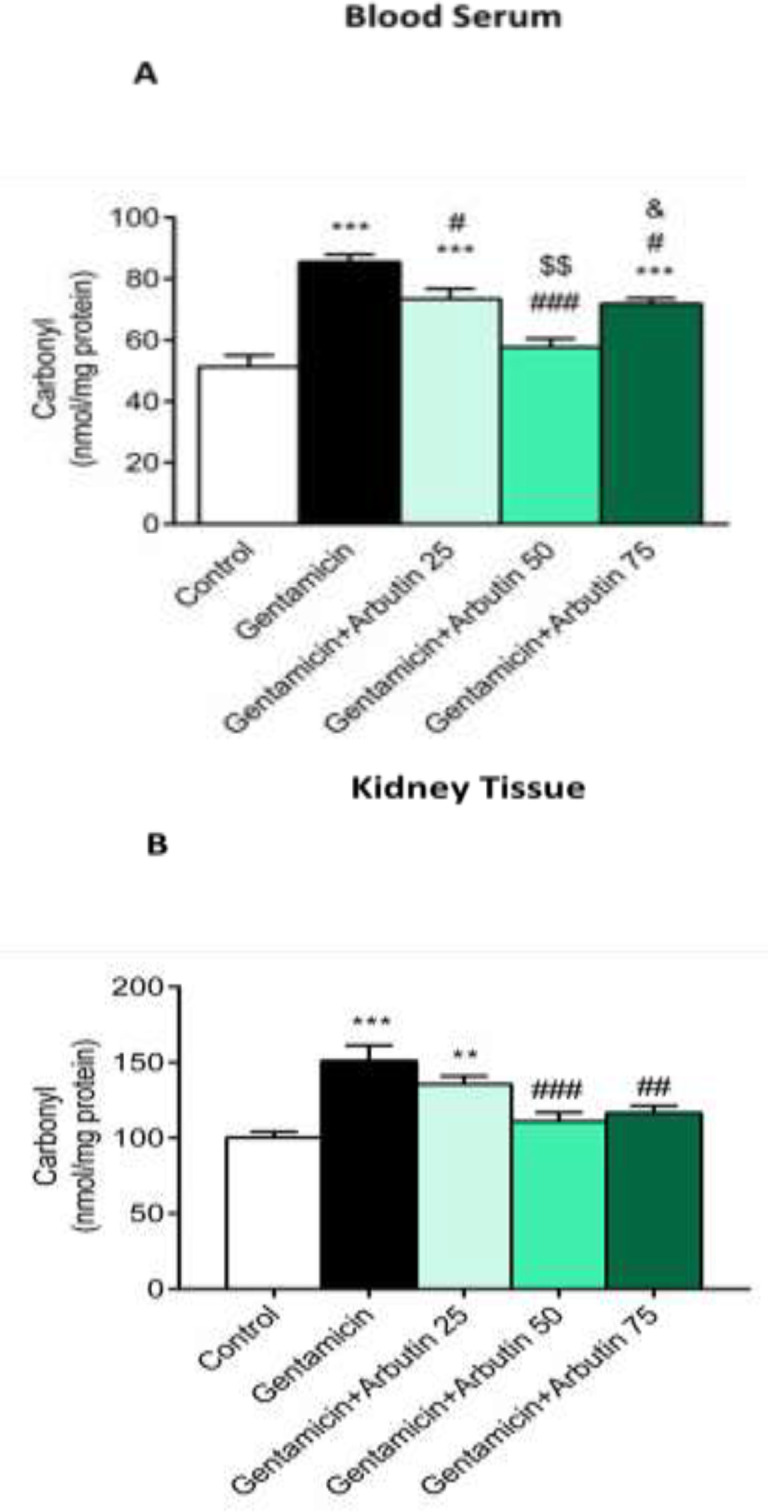
Effect of arbutin on serum and renal carbonyl levels in GM-induced nephrotoxicity

^**^p<0.01 and ^***^p<0.001 compared to the control; ^#^p<0.05, ^##^p<0.01 and ^###^p<0.001 compared to the GM group; ^$$^p<0.01 compared to the GM+arbutin 25 mg/kg and ^&^p<0.05 compared to the GM+arbutin 75 mg/kg. n=8. GM: Gentamicin.

Serum carbonyl level significantly increased in the group that received GM only (p<0.001) and the groups GM+arbutin (25 mg/kg, p<0.001) and GM+arbutin (75 mg/kg, p<0.001) compared to the control group. This level was significantly decreased in the GM+arbutin (50 mg/kg) compared to rats only treated with GM (p<0.001) and the groups GM+arbutin (25 mg/kg) and 75 mg/kg (p<0.05). A significant difference was also found in serum carbonyl level between GM+arbutin (50 mg/kg) and GM+arbutin (75 mg/kg) (p<0.05) (Figure 3A). Additionally, carbonyl level in kidney tissue significantly increased in the GM (p<0.001) and GM+arbutin group (25 mg/kg) (p<0.01) compared to the control group. Arbutin at doses of 50 (p<0.001) and 75 mg/kg (p<0.01) significantly decreased the carbonyl level in renal tissue compared to the GM group. However, GM+arbutin (25 mg/kg) had no significant difference compared to the GM treated rats (p =0.4367) (Figure 3B).

**Figure 4:**
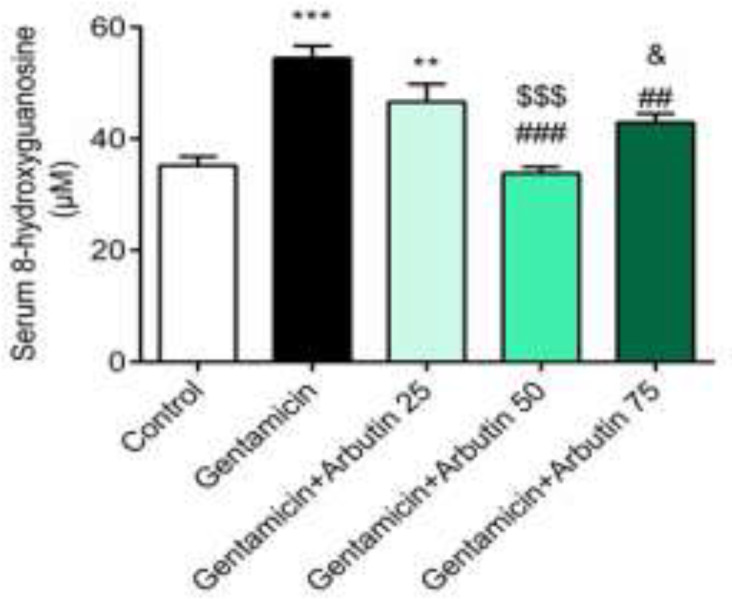
Effect of arbutin on serum 8-hydroxyguanosine level in GM-induced nephrotoxicity

^ ***^p<0.001 compared to the control; ^##^p<0.01 and ^###^p<0.001 compared to the GM group; ^$$$^p<0.001 compared to the GM+arbutin 25 mg/kg and ^&^p<0.05 compared to the GM+arbutin 75 mg/kg. n=8. GM: Gentamicin.

Serum 8-hydroxyguanosine was remarkably increased in the GM (p<0.001) and GM+arbutin 25 mg/kg group (p<0.01) compared to the control group, while the increase of 8-hydroxyguanosine was not significant in the groups treated with GM+arbutin 50 mg/kg (p =0.9884) and 75 mg/kg (p =0.0874) compared to the control group. Serum 8-hydroxyguanosine levels had significant changes in the GM+arbutin 50 mg/kg (p<0.001) and 75 mg/kg (p<0.01) groups. In addition, there was a significant difference in serum 8-hydroxyguanosine between GM+arbutin (50 mg/kg) and GM+arbutin 25 mg/kg (p<0.001) and GM+arbutin 75 mg/kg (p<0.05) (Figure 4).

**Effects of arbutin on TBARS and FRAP levels in GM-induced nephrotoxicity**

**Figure 5:**
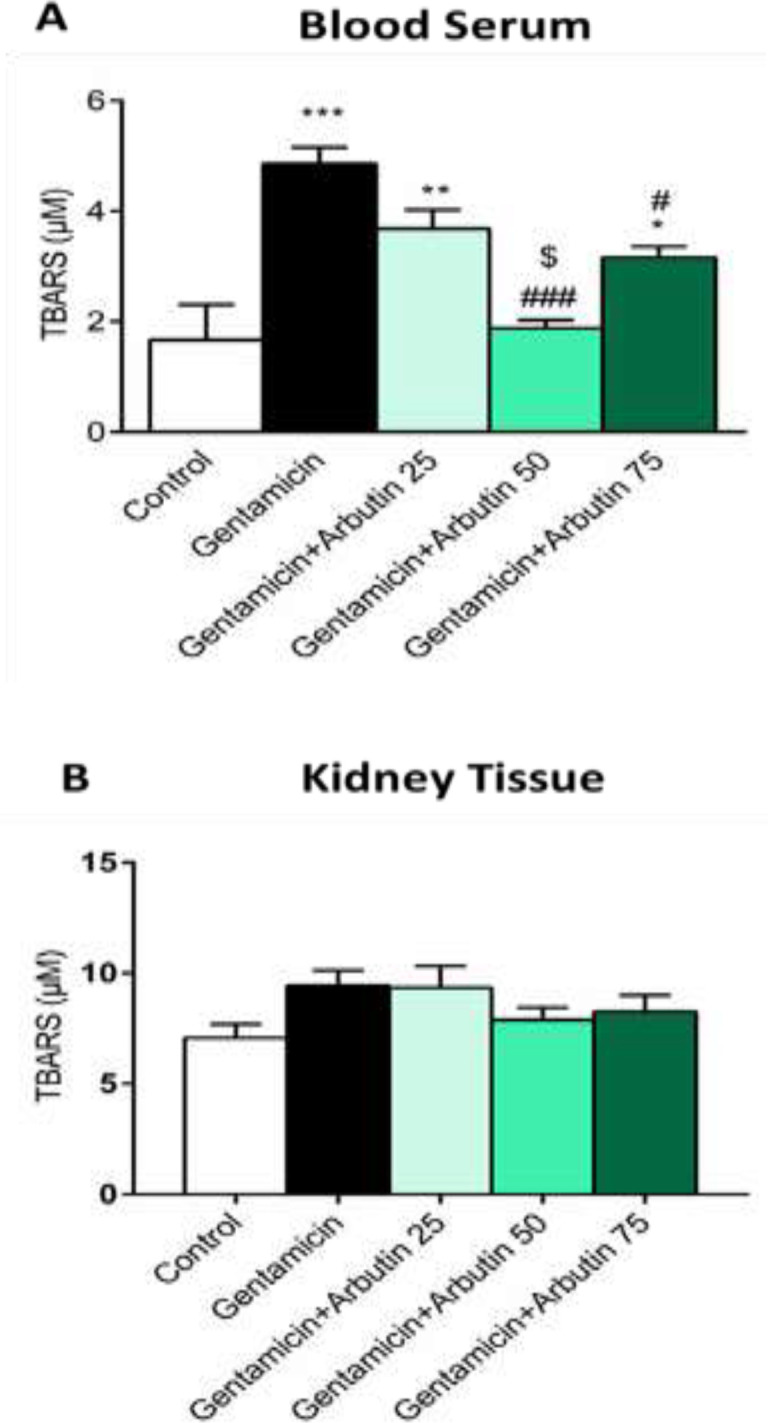
Effect of arbutin on TBARS level in GM-induced nephrotoxicity

^*^p<0.05,^ **^p<0.01 and ^***^p<0.001 compared to the control; ^#^p<0.05 and ^###^p<0.001 compared to the GM group and ^$^p<0.05 compared to the GM+arbutin 25 mg/kg. n=8.

The results presented that TBARS level enhanced in the GM only treated group (p<0.001) and GM+arbutin 25 (p<0.01) and 75 mg/kg (p<0.05) groups compared to the control. A significant difference in TBARS level was found between GM+arbutin 50 mg/kg (p<0.001) and GM+arbutin 75 mg/kg compared to the GM group (p<0.05) (Figure 5A). Renal TBARS levels showed no significant changes in all experimental groups (Figure 5B).

**Figure 6:**
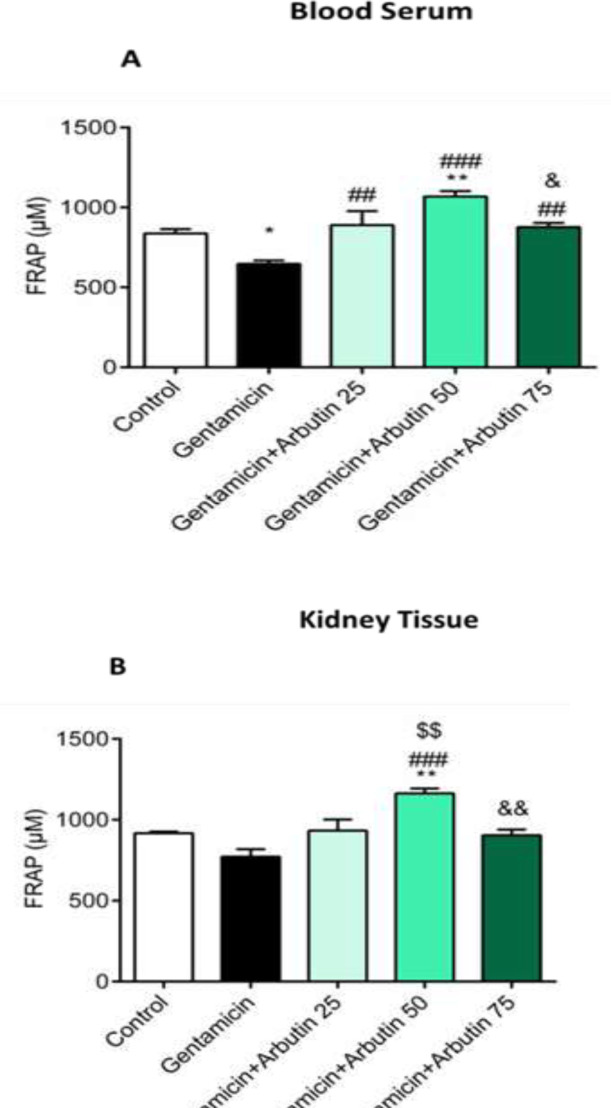
Effect of arbutin on FRAP level in GM-induced nephrotoxicity

^*^p<0.05 and^ **^p<0.01 compared to the control; ^##^p<0.01 and ^###^p<0.001 compared to the GM group and ^$$^p<0.01 compared to the GM+arbutin 25 mg/kg; ^&^p<0.05 and ^&&^p<0.01 compared to the GM+arbutin 75 mg/kg. n=8. GM: Gentamicin.

Serum level of total antioxidants (FRAP) had a significant reduction in GM only treated group (p<0.05) and GM+arbutin 50 mg/kg groups in comparison to the control group (p<0.01). A significant increase in FRAP level was found between arbutin-treated animals and GM only group (arbutin 25 mg/kg: p<0.01; arbutin 50 mg/kg: p<0.001; and arbutin 75 mg/kg: p<0.01). In addition, serum level of FRAP significantly increased in GM+arbutin 50 mg/kg compared to the GM+arbutin 75 mg/kg (p<0.05) (Figure 6A). 

The FRAP level in renal tissue was significantly increased in GM+arbutin 50 mg/kg compared to the control group (p<0.01). Administration of arbutin 50 mg/kg significantly increased the FRAP level in renal tissue compared to the GM-treated rats (p<0.001). A significant difference in FRAP level was found between GM+arbutin 50 mg/kg and GM+arbutin 25 mg/kg (p<0.01) and 75 mg/kg (p<0.01) (Figure 6B).

**Effects of arbutin on histopathology of kidney**

**Figure 7:**
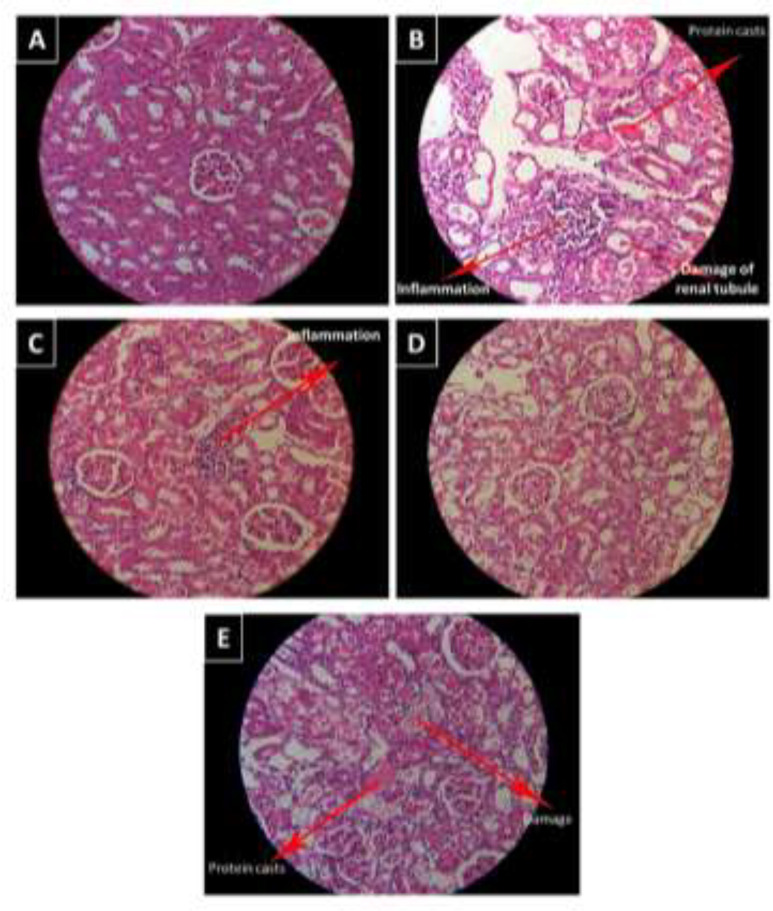
Arbutin attenuated degradation of renal tubules in GM-induced nephrotoxicity. (A) Control, (B) Gentamicin (GM), (C), (D) and (E) Groups receiving GM+arbutin at doses of 25, 50, 75 mg/kg, respectively. Magnification 40X

In the group receiving saline, kidney tissue had normal structure and no certain pathological lesion was observed (Figure 7A). In contrast, histological changes including numerous inflammatory cells, cast of proteins in renal tubules, degradation of proximal and distal tubules, were found in the GM group (Figure 7B). There were inflammatory cells and degradation of renal tubules in GM+arbutin 25 and 50 mg/kg (Figure 7C and E). Interestingly, in the GM+arbutin 50 mg/kg-treated rats, renal tubules were almost normal and less damage to kidneys were observed (Figure 7D). 

## Discussion

The results indicated that simultaneous application of GM and arbutin, especially at the dose of 50 mg/kg, prevents GM-induced nephrotoxicity in rats. The protective effect of arbutin might be partly mediated via inhibition of lipid peroxidation and antioxidant properties of arbutin. 

It has been shown that GM is actively reabsorbed in the proximal tubule of kidney and its concentration in tubular cells impairs the blood circulation in the kidneys which decreases the glomerular filtration rate and subsequently increases levels of plasma creatinine and blood urea nitrogen (Ghaznavi and Kadkhodaee, 2007 ▶). The creatinine level has a direct relation with the level of kidney damage and impaired renal function and in fact, it is a criterion for renal and proximal tubule function. In the current study, these indices significantly decreased in animal groups which received arbutin with GM. 

Nasri et al. showed that creatinine and urea levels were decreased in animals receiving the ethanolic extract of garlic compared to GM group (Nasri et al., 2013 ▶). Curcumin also reduced the acute kidney injury through attenuation of oxidative stress and apoptosis of renal tubular cells (He et al., 2015 ▶). Therefore, it seems that the renal protective effects of arbutin can be attributed to its strong antioxidant activity. 

Our previous studies revealed the effective role of arbutin in enhancement of antioxidant capacity. It has been shown that arbutin decreases the serum levels of lipid peroxidation and increases the antioxidant capacity in cyclosporine-induced toxicity (Khadir et al., 2015 ▶). Furthermore, arbutin reduced the behavioral impairments through attenuation of oxidative stress in experimental model of Parkinson's disease (Dadgar et al., 2018 ▶). Administration of arbutin also attenuated memory impairment and decreased the serum and hippocampal levels of oxidative and nitrosative stress in an AD animal model (Dastan et al., 2019 ▶). Further study indicated that arbutin reduced the seizure-related behaviors and ameliorated glial activation in an animal model of epilepsy (Ahmadian et al., 2019 ▶). 

In line with previous reports, application of arbutin at the dose of 50 mg/kg was more effective compared to doses 25 or 75 mg/kg. Khadir et al. suggested that administration of arbutin at the dose of 50 mg/kg led to a protective effect against cyclosporine-induced toxicity, while meaningful oxidative and lipoperoxidative activities were found in rats that were treated with a high dose of arbutin (100 mg/kg) (Khadir et al., 2015 ▶). Interestingly, Baradaran et al. also demonstrated that lower dose of hesperetin as a natural compound, decreased hippocampal oxidative stress level, while treatment with high doses of hesperetin increased the lipid peroxidation index (Baradaran et al., 2018 ▶). In spite of antioxidant effects of some natural products, it has been shown that administration of such compounds at high doses may increase toxicity and oxidative stress (Bouayed and Bohn, 2010 ▶). 

In conclusion, our data indicated that administration of arbutin reduces the GM-induced nephrotoxicity. The renal protective impact of arbutin is partly mediated by decreasing the peroxidation of lipids, proteins and nucleic acids.

## References

[B1] Ahmadian SR, Ghasemi-Kasman M, Pouramir M, Sadeghi F (2019). Arbutin attenuates cognitive impairment and inflammatory response in pentylenetetrazol-induced kindling model of epilepsy. Neuropharmacol.

[B2] Ali B (2003). Agents ameliorating or augmenting experimental gentamicin nephrotoxicity: Some recent research. Food Chem Toxicol.

[B3] Banday AA, Farooq N, Priyamvada S, Yusufi AN, Khan F (2008). Time dependent effects of gentamicin on the enzymes of carbohydrate metabolism, brush border membrane and oxidative stress in rat kidney tissues. Life Sci.

[B4] Baradaran S, Ghasemi-Kasman M, Ebrahimpour A, Ahmadian S, Pouramir M (2018). Anticonvulsant effects of hesperetin in animal model of pentylenetetrazole-induced-seizures. J Babol Univ Medical Sci.

[B5] Boroushaki MT, Fanoudi S, Mollazadeh H, Boroumand-Noughabi S, Hosseini A (2019). Reno-protective effect of rheum turkestanicum against gentamicin-induced nephrotoxicity. Iran J Basic Med Sci.

[B6] Boroushaki MT, Sadeghnia HR (2009). Protective effect of safranal against gentamicin-induced nephrotoxicity in rat. IJBMS.

[B7] Bouayed J, Bohn T (2010). Exogenous antioxidants—double-edged swords in cellular redox state: Health beneficial effects at physiologic doses versus deleterious effects at high doses. Oxid Med Cell Longev.

[B8] Cao L, Zhi D, Han J, Kumar Sah S, Xie Y (2019). Combinational effect of curcumin and metformin against gentamicin‐induced nephrotoxicity: Involvement of antioxidative, anti‐inflammatory and antiapoptotic pathway. J Food Biochem.

[B9] Capasso G, Di Gennaro CI, Ragione FD, Manna C, Ciarcia R, Florio S, Perna A, Pollastro RM, Damiano S, Mazzoni O, Galletti P, Zappia V (2008). In vivo effect of the natural antioxidant hydroxytyrosol on cyclosporine nephrotoxicity in rats. Nephrol Dialy Transplant.

[B10] Dadgar M, Pouramir M, Dastan Z, Ghasemi-Kasman M, Ashrafpour M, Moghadamnia AA, Khafri S, Pourghasem M (2018). Arbutin attenuates behavioral impairment and oxidative stress in an animal model of parkinson's disease. Avicenna J Phytomed.

[B11] Dastan Z, Pouramir M, Ghasemi-Kasman M, Ghasemzadeh Z, Dadgar M, Gol M, Ashrafpour M, Pourghasem M, Moghadamnia AK, Khafri S (2019). Arbutin reduces cognitive deficit and oxidative stress in animal model of alzheimer's disease. Int J Neurosci.

[B12] Farombi E, Ekor M (2006). Curcumin attenuates gentamicin-induced renal oxidative damage in rats. Food Chem Toxicol.

[B13] Farooq N, Priyamvada S, Khan F, Yusufi A (2007). Time dependent effect of gentamicin on enzymes of carbohydrate metabolism and terminal digestion in rat intestine. Hum Exp Toxicol.

[B14] Ghaznavi R, Kadkhodaee M (2007). Comparative effects of selective and non-selective nitric oxide synthase inhibition in gentamicin-induced rat nephrotoxicity. Arch Toxicol.

[B15] Hasanvand A, Kharazmkia A, Mir S, Khorramabadi RM, Darabi S (2018). Ameliorative effect of ferulic acid on gentamicin-induced nephrotoxicity in a rat model; role of antioxidant effects. J Renal Inj Prev.

[B16] He L, Peng X, Zhu J, Liu G, Chen X, Tang C, Liu H, Liu F, Peng Y (2015). Protective effects of curcumin on acute gentamicin-induced nephrotoxicity in rats. Can J Physiol Pharmacol.

[B17] Khadir F, Pouramir M, Joorsaraee SG, Feizi F, Sorkhi H, Yousefi F (2015). The effect of arbutin on lipid peroxidation and antioxidant capacity in the serum of cyclosporine-treated rats. Caspian J Intern Med.

[B18] Mingeot-Leclercq M-P, Glupczynski Y, Tulkens PM (1999). Aminoglycosides: Activity and resistance. Antimicrob Agents Chemother.

[B19] Nagai J, Takano M (2004). Molecular aspects of renal handling of aminoglycosides and strategies for preventing the nephrotoxicity. Drug Metab Pharmacok.

[B20] Nasri H, Nematbakhsh M, Rafieian-Kopaei M (2013). Ethanolic extract of garlic for attenuation of gentamicin-induced nephrotoxicity in wistar rats. Iran J Kidney Dis.

[B21] Shahaboddin M-E, Pouramir M, Moghadamnia A-A, Parsian H, Lakzaei M, Mir H (2011). Pyrus biossieriana buhse leaf extract: An antioxidant, antihyperglycaemic and antihyperlipidemic agent. Food Chem.

[B22] Taha MME, Salga MS, Ali HM, Abdulla MA, Abdelwahab SI, Hadi AHA (2012). Gastroprotective activities of turnera diffusa willd Ex schult Revisited: Role of arbutin. J Ethnopharmacol.

[B23] Yousefi F, Mahjoub S, Pouramir M, Khadir F (2013). Hypoglycemic activity of pyrus biossieriana buhse leaf extract and arbutin: Inhibitory effects on alpha amylase and alpha glucosidase. Caspian J Intern Med.

